# Disease of Adrenal Glands

**DOI:** 10.1155/2015/403521

**Published:** 2015-03-26

**Authors:** Gianluca Iacobellis, Gian Paolo Rossi, Frederic Castinetti, Claudio Letizia

**Affiliations:** ^1^Division of Endocrinology, Diabetes and Metabolism, Department of Medicine, University of Miami Miller School of Medicine, Miami, FL 33136, USA; ^2^Department of Medicine (DIMED), Clinica dell'Ipertensione Arteriosa, University Hospital, University of Padua, 31528 Padua, Italy; ^3^Department of Endocrinology, La Timone Hospital, Hopitaux de Marseille and Centre de Recherche en Neurobiologie et Neurophysiologie de Marseille, Aix-Marseille University, 13385 Marseille, France; ^4^Secondary Hypertension Unit, Department of Internal Medicine and Medical Specialties, Sapienza University of Rome, 00165 Rome, Italy

The adrenal gland has been historically an object of interest and scientific curiosity. This is also due to its very heterogeneous structure, number of hormones, complex neural innervation, and multiple and different physiological functions. The adrenal gland also entails an outstanding example of paracrine interactions occurring between histogenetically different tissues as the cortex and the medulla.

This special issue is a great opportunity for the reader to learn the latest and emerging findings on the pathophysiology, diagnosis, and treatment of the adrenal glands disorders. The issue provides a variety of excellent articles covering a broad and contemporary spectrum of aspects of the diseases of the adrenal gland.

Of particular interest and novelty is the interplay between hormones of the adrenal glands and other organs, such as the adipose tissue, the endothelium, the bone, and even the brain. In addition to the well-established effects on lipid and glucose metabolism, the hormones of the adrenal glands display a fascinating cross talk with the adipose tissue [1–3]. The interaction between the adrenals and adipokines is extensively discussed by A. Y. Kargi and G. Iacobellis in a comprehensive and updated review paper. The potential of the fat depot surrounding the adrenal tumors to act like a brown adipose tissue (BAT) is a rapidly emerging topic that will certainly deserve further attention and investigation [4]. Interestingly the authors provided a theoretical basis for potential future pharmacological interventions aimed at adrenal hormone targets in the adipose tissue.

Primary aldosteronism is the most common endocrine cause of arterial hypertension. It can cause excess damage to the organs that are target of hypertension and higher cardiometabolic risk [5]. This contention was supported by previous experimental data obtained by Karl Weber's group in rats infused with aldosterone, which exhibited hypercalciuria and raised parathyroid hormone (PTH) levels [6], and, more importantly, by findings in patients with aldosterone-producing adenoma who also showed elevated serum PTH levels that were then normalized by adrenalectomy [7]. The effect of the adrenal hormones on bone metabolism in patients with primary aldosteronism is nicely addressed by L. Petramala et al. The authors sought to test the hypothesis that hyperaldosteronism may influence mineral homeostasis through higher urinary calcium excretion leading eventually to secondary hyperparathyroidism. Of further interest, G. Mazzocchi et al. showed that PTH stimulates aldosterone secretion in a concentration-dependent manner [8], a finding that was complemented by the demonstration of the mineralocorticoid receptor in the human parathyroid cells [9]. It is well established that a substantial amount of sodium is bound to proteoglycans of bone, connective tissue, and cartilage and that the osmotic force created by the high sodium concentration maintains the high water content in the latter tissue, allowing it to withstand high pressures during exercise [10]. In this special issue P. Alonso et al. further expand on the relationships between the adrenal gland and the skeleton by showing a reduction in the bone mineral density in children with congenital adrenal hyperplasia when bone age rather than chronological age is considered. Altogether these findings further enhance the multitargeting effects of hormones like cortisol and aldosterone and open new avenues for the understanding of the interactions between adrenal and other hormones and tissues originally thought to be totally unrelated. These evidences are rapidly changing the paradigm that hormonal systems work independently.

V. Salpietro et al. extensively discussed the potential neurological manifestations in children with adrenal dysfunction and the compelling need for an early diagnosis.

The causative and prognostic role of neoangiogenesis in patients with pheochromocytoma is analyzed by M. Bialas and colleagues. Neoangiogenesis was evaluated by assessing microvessel density (MVD) and the expression of vascular endothelial growth factors (VEGFs). The study showed that MVD is not able to differentiate between benign and malignant pheochromocytomas. However, the authors proposed that high MVD could be a promising factor for antiangiogenic therapy in malignant cases of pheochromocytoma.

Accurate genetic testing of adrenal glands tumors, such as pheochromocytomas, is the topic of interesting articles by D. A. Rowbotham et al. and R. Martins and M. J. Bugalho. Promising results suggest that mutations in the von Hippel-Lindau (VHL) gene elongin BC protein complex could be important factor for the development of pheochromocytomas.

Genetic analysis is certainly a key factor, but how do we manage cardiovascular disease prevention in adrenal diseases? Cushing's syndrome and even subclinical hypercortisolism are associated with higher cardiovascular risk [11]. But do we have effective tools to predict and possibly prevent this risk? Y.-F. Wang et al. stressed out the need of using the congestive heart failure (C), hypertension (H), age (A), diabetes (D), and stroke (S), the CHADS2 score, to predict major cardiovascular events in patients affected by Cushing's syndrome. Perhaps, other biomarkers, such as imaging of organ-specific fat depot, will emerge for the cardiovascular risk stratification in the adrenal gland diseases [11].

Finally, the best surgical approach and treatment for different clinical conditions and adrenal tumors, such as adrenal benign tumors, incidentalomas, or metastatic adrenal tumors, are also object of discussion in this special issue. There is a consensus that laparoscopic adrenalectomy should be considered as the first choice treatment for the resection of adrenal benign tumors, as suggested by S. Chuan-yu et al, whereas the role of laparoscopic surgery in small incidentaloma is still controversial, as nicely discussed by M. Pędziwiatr et al. The surgical management of adrenal tumors is object of recent debate and investigation. A recent and large multinational observational retrospective population-based study suggested that adrenal-sparing surgery should be the surgical approach of choice for multiple endocrine neoplasia type 2-related phaeochromocytoma to reduce the Addisonian-like complications and consequent lifelong dependency on steroids [12].

In conclusion, the interest in the adrenal gland is now encompassing a broader audience of physicians and researchers. This special issue is aimed at engaging the reader in an exciting reading of the new insights on the adrenal glands and related disorders.

*Gianluca  Iacobellis* *Gianluca  Iacobellis* *Gian  Paolo  Rossi* *Gian  Paolo  Rossi* *Frederic  Castinetti* *Frederic  Castinetti* *Claudio  Letizia* *Claudio  Letizia*
